# Physicochemical compatibility of highly-concentrated solvate ionic liquids and a low-viscosity solvent†

**DOI:** 10.1039/c9ra04797b

**Published:** 2019-08-12

**Authors:** Keitaro Takahashi, Yuki Ishino, Wataru Murata, Yasuhiro Umebayashi, Seiji Tsuzuki, Masayoshi Watanabe, Hiromitsu Takaba, Shiro Seki

**Affiliations:** Department of Environmental Chemistry and Chemical Engineering, School of Advanced Engineering, Kogakuin University 2665-1 Nakano-machi Hachioji Tokyo 192-0015 Japan shiro-seki@cc.kogakuin.ac.jp +81-42-628-4568 +81-42-628-4568; Graduate School of Science and Technology, Niigata University 8050, Ikarashi, 2-No-cho, Nishi-ku, Niigata-shi Niigata 950-2181 Japan; National Institute of Advanced Industrial Science and Technology (AIST) 1-1-1 Umezono Tsukuba-shi Ibaraki 305-8568 Japan; Department of Chemistry and Biotechnology, Yokohama National University 79-5 Tokiwadai, Hodogaya-ku Yokohama Kanagawa 240-8501 Japan

## Abstract

High ionic carrier mobilities are important for the electrolyte solutions used in high-performance batteries. Based on the functional sharing concept, we fabricated mixed electrolytes consisting of solvate ionic liquids (SIL), which are highly concentrated solution electrolyte, and the non-coordinating low-viscosity dilution solvent 1,1,2,2-tetrafluoroethyl 2,2,3,3-tetrafluoropropyl ether (HFE). We investigated the thermal, transport, and static properties of electrolytes with different ratios of SIL to HFE. In particular, the interactions between the SILs and HFE and static correlations of the coordinating (ether-based molecules), non-coordinating (HFE), and carrier ionic species (lithium salt) were clarified by applying the excess density concept. Ether molecules always formed strong complexes with lithium cations regardless of the absence or presence of HFE. The repulsion force between the SILs and HFE was strongly affected by lithium salt concentration. From our results, we proposed dissociation/association models for these electrolyte systems.

## Abstract 2

Highly-concentrated and low viscosity electrolyte for Li–S batteries.

## Graphical abstract

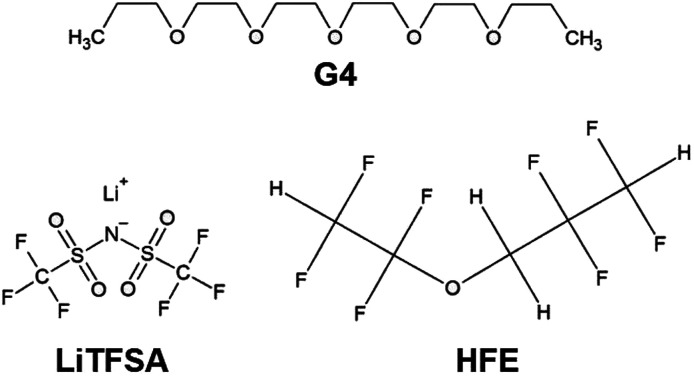

## 1. Introduction

Renewable energy technology or industry such as solar, wind power, geothermal heat, and biomass are prospective contributors to achieve a sustainable low-carbon society because they do not emit greenhouse gases.^1^ However, renewable energies are unstable because of their large output fluctuations and both their power generation and equipment are expensive. To solve these problems, it is necessary to develop large-scale secondary battery systems to level the input and output loads of natural energies.^2–5^ Renewable energy also needs to meet the demands of low cost, high energy density, and long life to realize its effective use. Lithiumsulfur (Li–S) batteries are attracting attention as possible successors of Li-ion batteries with high-energy density and low cost.^6^ Generally, Li–S batteries are composed of an S/carbon composite as a positive electrode, Li metal as the negative electrode, and mixture of solvent(s) and salt(s) as electrode as the electrolyte.^7^ An S positive electrode has a larger theoretical capacity (1672 mA h g^−1^) than that of conventional electrode materials such as Li_*x*_CoO_2_ (0.5 < *x* < 1; 137 mA h g^−1^). Elemental S (S_8_) can react in multistep processes (discharge: cleavage, charge: recombination) involving 16 electron transfer reactions. These properties make Li–S battery systems promising as innovative high-energy-density power storage devices. In addition, elemental S is environmentally friendly, inexpensive because it is obtained as a by-product from oil refining, and also has low toxicity, which makes it a safe positive electrode material. However, the reaction intermediate lithium polysulfide (Li_2_S_*x*_; *x* = 2–8) can dissolve into commonly used electrolyte solution during charge/discharge at the positive electrode/electrolyte interface, which leads to cell degradation during cycling.^8–11^ Dissolution and diffusion of Li_2_S_*x*_ into the electrolyte solution has serious effects on the performances of Li–S batteries for the following reasons.

(1) Capacity degradation of the S electrode by decreasing of the net positive S electrode mass.

(2) Reduction reactions of dissolved Li_2_S_*x*_ (*x* = 1–2) at the Li metal negative electrode.

(3) Re-oxidative reaction of the reduced intermediate (generated as shown in (2)) at the S positive electrode (redox shuttle mechanism)

Therefore, control of Li_2_S_*x*_ dissolution is essential to realize high-performance Li–S batteries.

The solubility of Li_2_S_*x*_ depends on electrolyte, and therefore the design of electrolyte is important for improving battery performance. Dokko *et al.* used solvate ionic liquids (SILs) as the electrolyte for Li–S batteries owing to the low solubility of Li_2_S_*x*_.^12^ Their SILs consisted of a 1 : 1 complex of glyme molecules and Li salt with weak Lewis acidity/basicity. Glyme molecules and Li cations can form quite stable coordinated cations, such as [Li(Glyme)]^+^, which exist in the liquid state at room temperature, and have weak basicity with similar complex structures.^13,14^ SILs are a type of room-temperature ionic liquid because of their liquid properties at room temperature and composition of complex cations and counter anions.^15^ As room-temperature ionic liquids, SILs show not only high thermal stability and low volatility but also high ionic conductivity and a wide electrochemical window.^13^ Meanwhile, SILs hardly interact with Li_2_S_*x*_, and can control the dissolution of the reactive intermediate Li_2_S_*x*_ into electrolyte solution.^12^ Long cycle life (over 800 cycles) and high coulombic efficiencies (over 97%) have been realized by using Li–S batteries.^16^ However, the glyme and Li salt are not equimolar near the electrode/electrolyte interface owing to the Li alloying and de-alloying into the electrode associated with charge/discharge process, even if the equimolar mixture is used for the electrolyte.^17^ Thus, Li_2_S_*x*_ can dissolve into the electrolyte through dynamic electrochemical reactions because of the presence of free glyme molecules^18^ originating from the desolvated SIL. Against such a background, we proposed using non-equimolar SILs (mixture of glyme with excess Li salt) to suppress battery degradation through the formation of robust complexes.^19^ Such an electrolyte can suppress the dissolution of Li_2_S_*x*_ into free glyme (non-coordinated solvent molecules) during the discharge process of Li–S batteries.^20^

Recently, ‘super-concentrated’ electrolytes using other solvent systems have attracted attention for use as chemically/electrochemically stable electrolytes.^21–24^ However, the viscosity of electrolyte systems increases with of Li salt concentration, which raises the internal resistance of the cell and decreases of input/output performance. To obtain a low-viscosity electrolyte solution, the concept of dilution with the low-viscosity non-coordinating fluorinated solvent 1,1,2,2-tetrafluoroethyl 2,2,3,3-tetrafluoropropyl ether (HFE) has been proposed to realize electrochemically stable batteries with suitable charge/discharge rate capability without destroying the solvated structure of SILs.^25^ In previous research, dilution of HFE was valid for systems with a 1 : 1 molar ratio of glyme to Li salt; however, the effects of dilution with HFE on non-equimolar SILs were unclear. Also, understanding the interactions between HFE and SILs (including the coordinated solvent and Li salt) is very important for designing of high-performance Li–S batteries. In this report, we investigate the thermal, static (density), and transport (viscosity) properties of mixture of SILs and HFE. The interactions between coordinated solvent (glyme) and non-coordinated solvent (HFE) molecules and dissolved salts are clarified.

## 2. Experimental

### 2.1 Samples

**Fig. 1:**
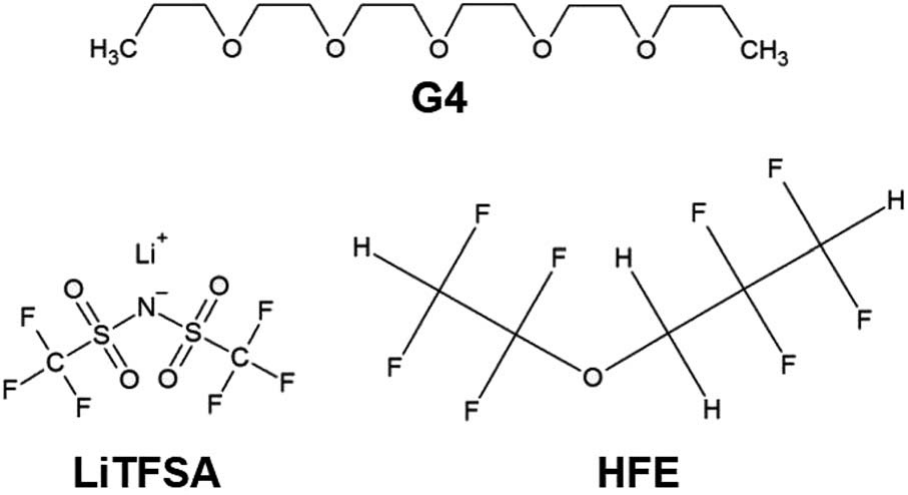
Chemical structures of G4, LiTFSA and HFE.

Distilled and dried tetraglyme (G4, Nippon Nyukazai Co., Ltd.; Fig. 1) and lithium bis(trifluoromethanesulfonyl)amide (LiN(SO_2_CF_3_)_2_, LiTFSA, Solvay Co., Ltd.; Fig. 1) were used as the solvent and lithium species, respectively. G4 and LiTFSA were used to prepare [Lia(G4)_1_]TFSA_*a*_ SILs. These materials were stored in a dry-argon-filled glovebox ([O_2_] < 1 ppm, [H_2_O] < 1 ppm, Miwa MFG Co., Ltd.). Molar ratios of G4 to LiTFSA of 1 : 1 and 1 : 1.25 were weighed and mixed to give homogeneous electrolytes [Li_1_(G4)_1_]TFSA, [Li_1.25_(G4)_1_]TFSA_1.25_, respectively. HFE (Daikin Industries Co., Ltd.; Fig. 1) was used as a solvent to dilute these electrolytes by a predetermined amount.

### 2.2 Thermal analysis

The thermal properties of prepared samples were investigated using a thermogravimetry/differential thermal analysis (TG/DTA) using a Thermo plus EVO2 analyzer (Rigaku) from 303.15 to 753.15 K at a heating rate of 10 K min^−1^. The thermal transitions of prepared samples were also investigated using the differential scanning calorimetry (DSC) on the same systems. Samples for DSC measurements were hermetically sealed in aluminium pans in a dry argon-filled glovebox. Thermograms were recorded during a cooling scan (303.15 K to 173.15 K) followed by a heating scan (173.15 to 303.15 K), at the same cooling and heating rates of 10 K min^−1^.

### 2.3 Viscosity and density measurements

Viscosity (*η*) and density (*ρ*) measurements were carried out using a Stabinger-type viscometer/density measurement system (SVM3000/G2, Anton Paar). The temperature was set in the range of 283.15 to 353.15 K at 5 K intervals while heating samples.

## 3. Results and discussion

### 3.1 Thermal properties

**Fig. 2:**
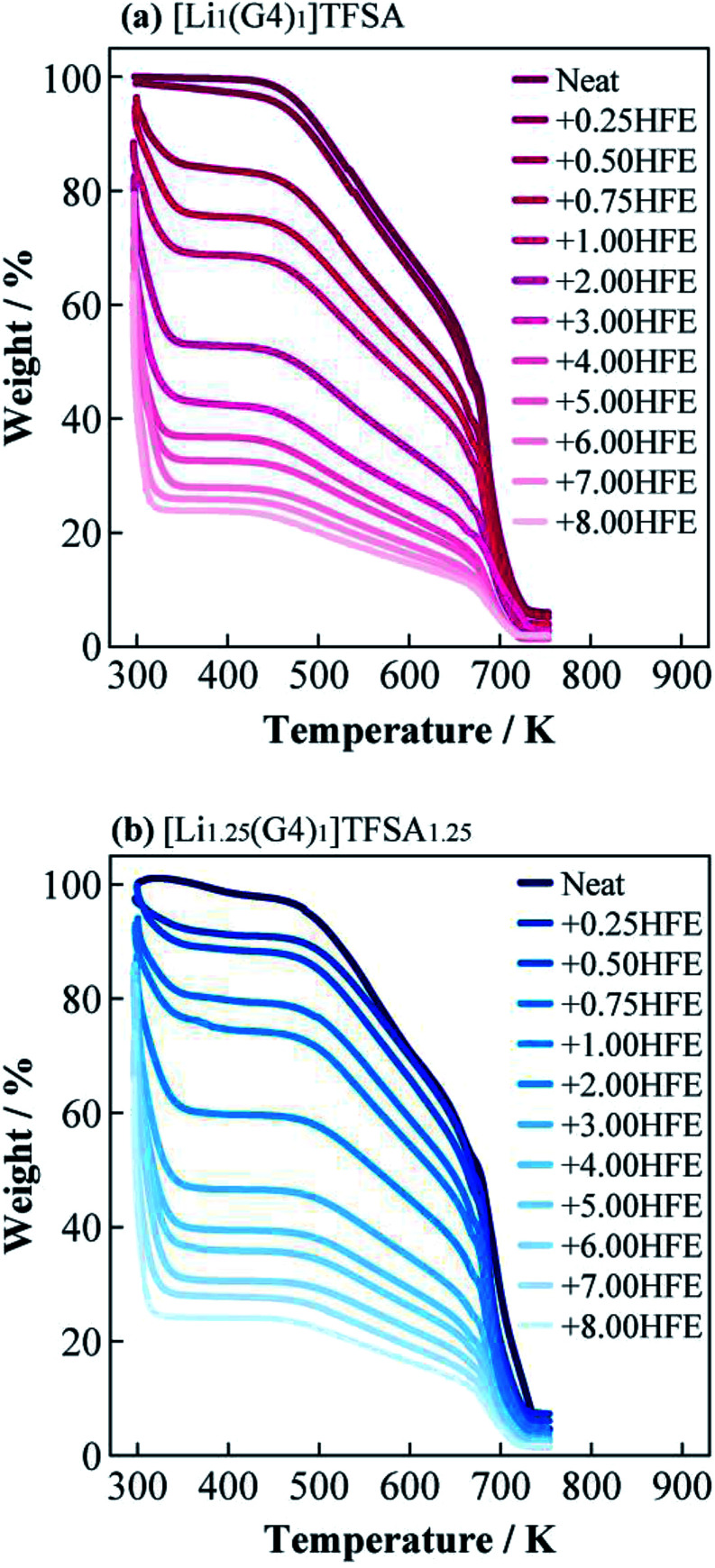
Thermogravimetric curves of (a) [Li_1_(G4)_1_]TFSA (a) and (b) [Li_1.25_(G4)_1_]TFSA_1.25_ diluted with HFE.

Fig. 2(a) and (b) shows the TG curves of [Li_1_(G4)_1_]TFSA and [Li_1.25_(G4)_1_]TFSA_1.25_ diluted with HFE, respectively. Two weight losses were observed for both systems, which were consistent with the boiling point of HFE (low temperature region) and decomposition point of [Li_*a*_(G4)_1_]TFSA_*a*_ (high temperature region). Observed TG results of weight ratios of SIL to HFE were consistent in both systems. This results suggest that the interactions between [Li_*a*_(G4)_1_]TFSA_*a*_ and HFE were weak because no coordination changes were observed.^25,26^

**Fig. 3:**
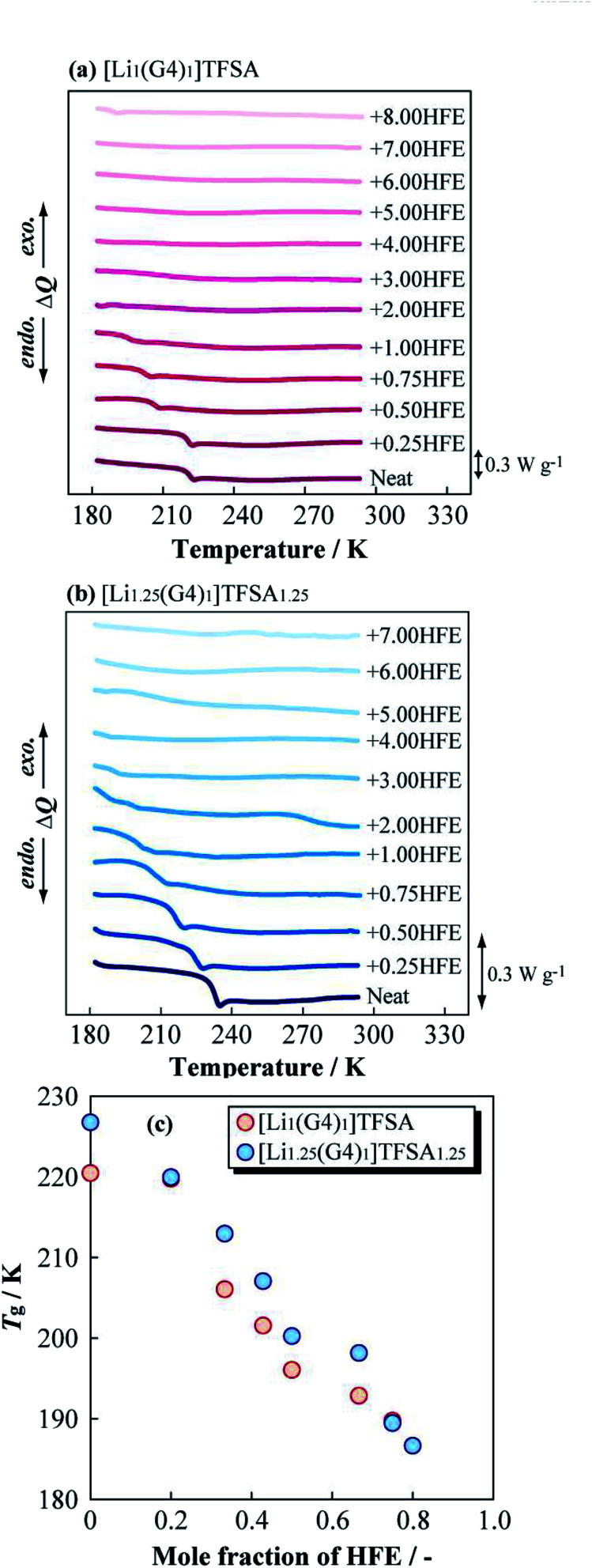
Differential scanning calorimetry curves of (a) [Li_1_(G4)_1_]TFSA and (b) [Li_1.25_(G4)_1_]TFSA_1.25_ diluted with HFE, and obtained glass transition temperatures (c).

Fig. 3(a) and (b) present DSC curves for the heating process of [Li_1_(G4)_1_]TFSA and [Li_1.25_(G4)_1_]TFSA_1.25_ diluted with HFE, respectively. The glass transition temperatures (*T*_g_) of [Li_1_(G4)_1_]TFSA and [Li_1.25_(G4)_1_]TFSA_1.25_ confirmed that the molar ratio of HFE to [Li_*a*_(G4)_1_]TFSA_*a*_ was less than one in both systems. Moreover, *T*_g_ values shifted to lower temperature with increasing HFE mole fraction, which was attributed to freezing point depression. Fig. 3(c) shows the relationship between observed *T*_g_ and HFE mole fraction for two systems. *T*_g_ of both [Li_1_(G4)_1_]TFSA and [Li_1.25_(G4)_1_]TFSA_1.25_ decreased by more than 30 K upon dilution with HFE.

### 3.2 Physical properties

**Fig. 4:**
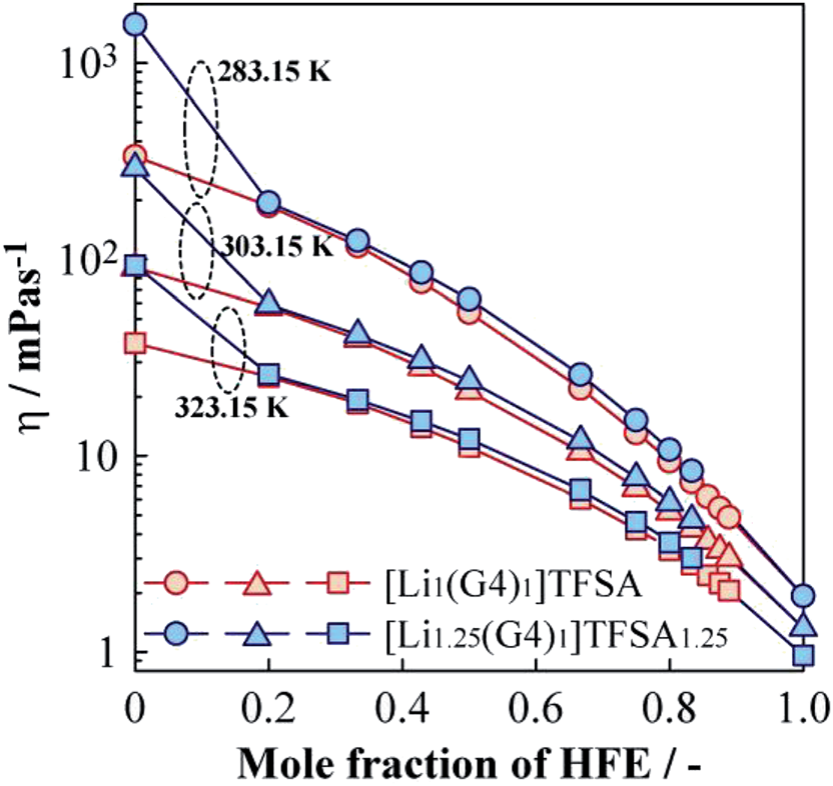
Molar fraction dependences of the viscosity for [Li_1_(G4)_1_]TFSA and [Li_1.25_(G4)_1_]TFSA_1.25_ diluted with HFE at 283.15, 303.15 and 323.15 K.

Fig. 4 displays the dependence of *η* of [Li_1_(G4)_1_]TFSA and [Li_1.25_(G4)_1_]TFSA_1.25_ on the molar fraction of HFE at 283.15, 303.15, and 323.15 K. For the neat SILs (mole fraction of HFE = 0), *η* was about 90 mPa s (*a* = 1, [Li_*a*_(G4)_1_]TFSA_*a*_) and 293 mPa s (*a* = 1.25) at 303.15 K; *i.e.*, [Li_1_(G4)_1_]TFSA was about 3 times less viscous than [Li_1.25_(G4)_1_]TFSA_1.25_. At all measured temperatures, the neat SIL with *a* = 1.25 displayed higher *η* values than those for the neat SIL with *a* = 1. This is because of difference in density caused by the number of cross-linking points of ether oxygen and Li^+^ reflecting LiTFSA concentration. In the case of the diluted samples (mole fraction of HFE > 0), a marked decrease in *η* was observed with increasing HFE content. Focusing on the relationship between HFE content and *η*, *η* of the samples with *a* = 1 samples showed a convex decreasing trend, and *η* values reached to those of neat HFE at all temperatures. In contrast, the samples with *a* = 1.25 exhibited marked decreases of *η* in the region between *x* = 0 and 0.2. Surprisingly, *η* values of the samples with *a* = 1.25 were almost the same as those for the samples with *a* = 1 even though the neat SILs have different *η* values. That is, the samples with *a* = 1 and 1.25 samples showed almost the same *η* values after HFE dilution, which means the fluidities of these electrolyte solutions would be the same. This result indicates that the excess LiTFSA (25%) in the samples *a* = 1.25 should not affect the bulk physicochemical parameters of these mixtures. More specifically, addition of a small amount of HFE should leads to the same liquid state regardless of LiTFSA concentration into the SIL. In addition, *η* values of diluted (+1.00 HFE) [Li_1_(G4)_1_]TFSA and [Li_1.25_(G4)_1_]TFSA_1.25_ samples were 21 and 24 mPa s at 303.15 K, respectively, which were considerably lower than those of the neat SILs. Therefore, dilution with HFE effectively improved the bulk transport properties of the SILs.

**Fig. 5:**
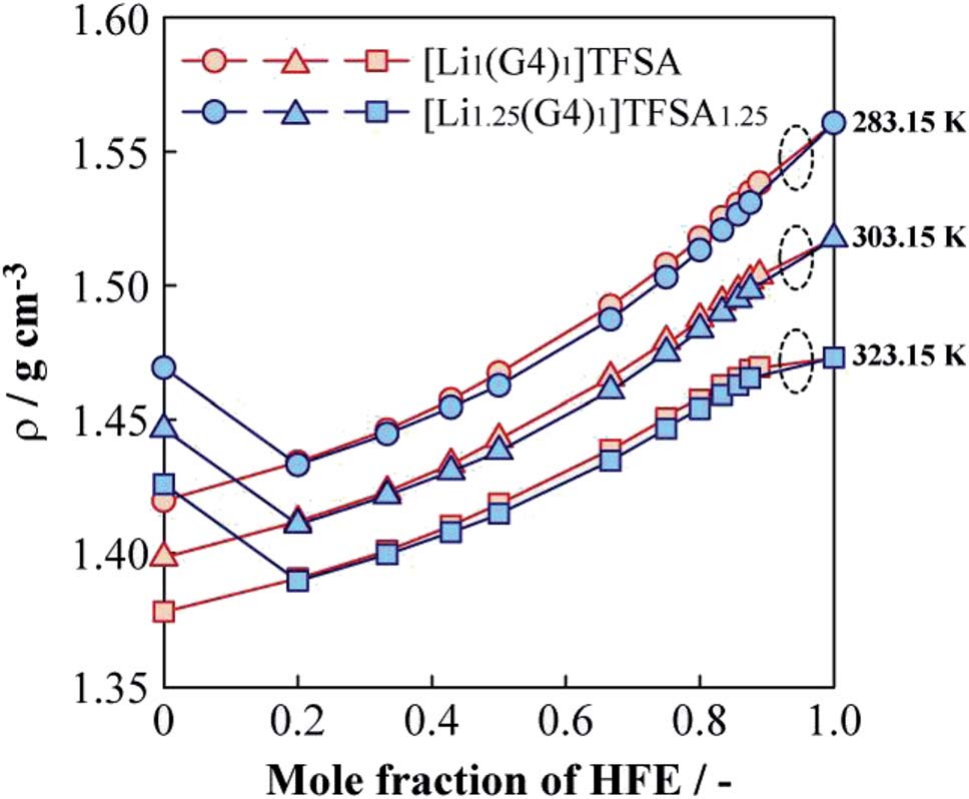
Molar fraction dependences of the density for [Li_1_(G4)_1_]TFSA and [Li_1.25_(G4)_1_]TFSA_1.25_ diluted with HFE at 283.15, 303.15 and 323.15 K.

Fig. 5 shows the relationships between the density (*ρ*) of [Li_1_(G4)_1_]TFSA and [Li_1.25_(G4)_1_]TFSA_1.25_ and mole fraction of HFE added at 283.15, 303.15 and 323.15 K. For the neat SILs, *ρ* were about 1.40 g cm^−3^ (*a* = 1) and 1.45 g cm^−3^ (*a* = 1.25) at 303.15 K; *ρ* of the sample with *a* = 1.25 was larger than that of the sample with *a* = 1. In general, *ρ* of electrolyte solutions depend on salt concentration because of the change of fluorine contents into electrolyte solution, which is consistent with our findings. Neat HFE displayed a higher *ρ* than that of neat [Li_*a*_(G4)_1_]TFSA_*a*_ (*a* = 1, 1.25), and *ρ* values generally increased upon addition of HFE. Considering the shift of *ρ versus* that of neat HFE, samples with *a* = 1 exhibited a convex increasing trend with increasing HFE content at all temperatures, with *ρ* values approaching that of neat HFE. On the other hand, observed *ρ* values of *a* = 1.25 samples decreased markedly between *x* = 0 and *x* = 0.2 at all temperatures, similar to the viscosity behaviour. In the region of *x* > 0.2, similar increasing tendencies of *ρ* were observed for both [Li_*a*_(G4)_1_]TFSA_*a*_ systems (slightly smaller *ρ* values were obtained for the samples with *a* = 1.25 than for those with *a* = 1, vide ante). This result suggests the possibility of achieving the independence of *ρ* and LiTFSA content by dilution with only a small amount of HFE. Moreover, *ρ* of both [Li_*a*_(G4)_1_]TFSA_*a*_ systems (*a* = 1, 1.25) crossed at *x* = 0.2 for all measured temperatures. Generally, *ρ* values of electrolyte solutions increase with salt concentration in the solvent. However, in this study, *ρ* values decreased with rising LiTFSA concentration following HFE addition (*x* ≦ 0.2).

**Fig. 6:**
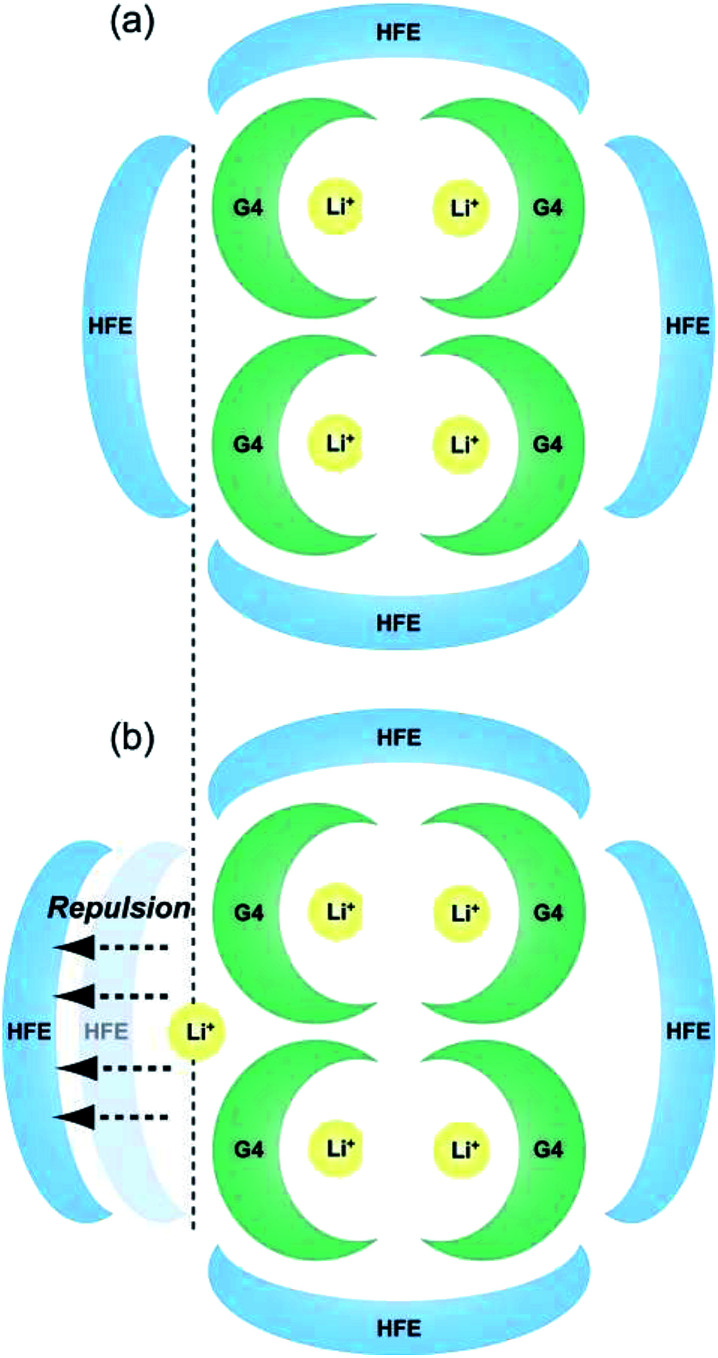
Schematic images of the proposed quasi-complex cation structure of (a) [Li_1_(G4)_1_]TFSA + HFE and (b) [Li_1.25_(G4)_1_]TFSA_1.25_ + HFE.

Schematic images of the quasi-complex cation structure of [Li_1_(G4)_1_]TFSA + HFE and [Li_1.25_(G4)_1_]TFSA_1.25_ + HFE are presented in Fig. 6(a) and (b), respectively. In static state, Li^+^ and G4 form stable complex cations with a 1 : 1 molar ratio and excess Li^+^ should promote repulsion with the dilution solvent HFE. In fact, LiTFSA and HFE cannot dissolve each other (Fig. S1†). As a result, *ρ* was decreased by addition of excess LiTFSA. Also, G4–Li^+^ complexes should always undergo ligand exchange and the diluted systems might form clusters consisting of SIL domains and HFE domains.

### 3.3 Interaction between SILs and HFE

The *ρ* values of [Li_*a*_(G4)_1_]TFSA_*a*_ (*a* = 1, 1.25) and HFE mixed electrolyte solutions were lower than the middle point between the *ρ* values of the neat SILs and HFE. To investigate the *ρ* changes upon mixing the SIL and HFE, the excess density (*E*_*ρ*_) was calculated as follows,^27^

**Fig. 7:**
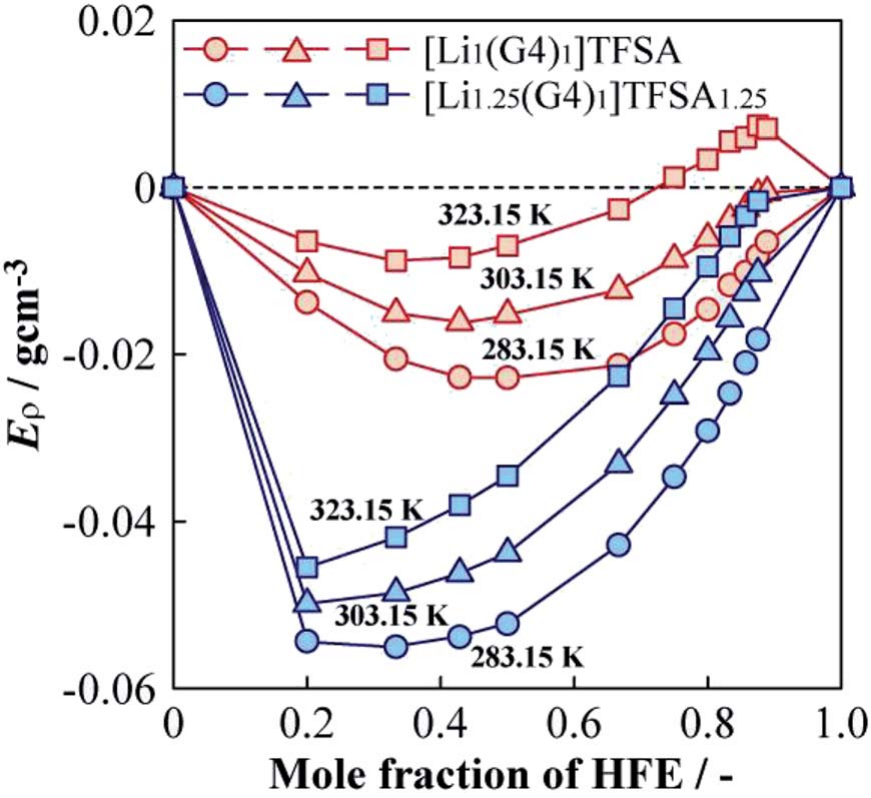
Molar fraction dependences of the excess density (*E*_*ρ*_) for [Li_1_(G4)_1_]TFSA and [Li_1.25_(G4)_1_]TFSA_1.25_ diluted with HFE at 283.15, 303.15 and 323.15 K.

1 *E*_*ρ*_ = *ρ*_measured_ − (*xρ*_HFE_ + (1 − *x*)*ρ*_SIL_)

where *x* is the molar fraction of HFE, *ρ*_measured_ is the measured density, *ρ*_HFE_ is the density of neat HFE, and *ρ*_SIL_ is the density of the neat SIL, respectively. Fig. 7 shows the dependences of *E*_*ρ*_ on HFE molar fraction for diluted [Li_1_(G4)_1_]TFSA and [Li_1.25_(G4)_1_]TFSA_1.25_ at 283.15, 303.15 and 323.15 K. The ideal line of *E*_*ρ*_ = 0 is indicated as a dotted line. Calculated *E*_*ρ*_ values were always negative values at temperatures ≤303.15 K, which are smaller than ideal, and *ρ* values were decreased by HFE dilution. These results imply that the SIL and HFE are immiscible in the microscopic sense; in other words, there is a repulsive force between them. Small (more negative) *E*_*ρ*_ values were always obtained for the samples with *a* = 1.25 than for those with *a* = 1, and the absolute values were considered as the degree of attractive or repulsive force. Therefore, the interaction between the SIL and HFE should be affected by the LiTFSA content of the SIL. The obtained results were correlated with the proposed electrolyte structures in Fig. 6. The decreasing *ρ* of the samples with *a* = 1.25 between *x* = 0 and 0.2 was consistent with the minimum *E*_*ρ*_ value at *x* = 0.2, which suggests the possibility of microphase separation of SIL and HFE. Our result were consistent with those of a previous spectroscopic report that indicated the formation of a microscopic stable coordination microstructure between Li^+^ and glyme molecules even if SIL was diluted with HFE.^25^ In contrast, positive values of *E*_*ρ*_ were observed for the samples with *a* = 1 and a high molar fraction of HFE at 323.15 K. In the case of high temperature and low SIL concentration, slight solubility between the SIL and HFE might occur through colligative property. In the future, we will further investigate the nanoscopic relationships between LiTFSA, G4 using high energy X-ray diffraction and Raman spectroscopy measurements.

## 4. Conclusions

Mixtures of solvated ionic liquids ([Li_1_(G4)_1_]TFSA, [Li_1.25_(G4)_1_]TFSA_1.25_) that were diluted with the solvent HFE were prepared, and their thermal, transport, and static properties were investigated. The results of this study are summarized as follows.

(1) The boiling temperature of HFE and decomposition temperature of SIL were observed independently in all measured systems. *T*_g_s of SILs were obtained regardless of HFE dilution even though freezing point depression was observed. Thermal investigations indicated there were no strong interactions between the SILs and HFE.

(2) The viscosity of [Li_1.25_(G4)_1_]TFSA_1.25_ was approximately three times higher than that of [Li_1_(G4)_1_]TFSA at 303.15 K. Upon dilution with HFE, the SILs possessed almost the same viscosity values independent of the Li salt concentration, which leads to equal fluidity in the electrolyte solutions. Dilution with HFE was therefore effective to achieve high ionic mobility of electrolyte solutions regardless of Li salt concentration.

(3) The densities of [Li_1.25_(G4)_1_]TFSA_1.25_ was higher than that of [Li_1_(G4)_1_]TFSA at all measured temperatures and the same tendencies were observed for density as for viscosity measurements. To analyze density changes of the mixtures caused by HFE dilution, excess densities were calculated as an index for the repulsion/attraction between the SILs and HFE. [Li_1.25_(G4)_1_]TFSA_1.25_ exhibited larger volume expansion than that of [Li_1_(G4)_1_]TFSA upon HFE dilution and the excess Li salt in the formed should increase the repulsion force of the SIL towards HFE. Therefore, compatibility of the SIL with excess Li salt and HFE is suitable from the viewpoint of battery performance, such as rate capability.

## Conflicts of interest

There are no conflicts of interest to declare.

## Supplementary Material

RA-009-C9RA04797B-s001
