# Supplementary material for: Physicochemical compatibility of highly-concentrated solvate ionic liquids and a low-viscosity solvent
Source: RSC Adv. 2019 Aug 12;9(43):24922–7. doi: 10.1039/c9ra04797b (PMC9069941; doi:10.1039/c9ra04797b)
Supplement: RA-009-C9RA04797B-s001 [file RA-009-C9RA04797B-s001.pdf]

Electronic Supplementary Material (ESI) for RSC Advances.
This journal is © The Royal Society of Chemistry 2019

Supporting Information

Physicochemical Compatibility of High-Concentrated Solvate Ionic Liquids and Low-viscosity Dilute Solvent

Keitaro Takahashi,[a] Yuki Ishino,[a] Wataru Murata,[a] Yasuhiro Umebayashi,[b] Seiji Tsuzuki,[c] Masayoshi Watanabe,[d] Hiromitsu Takaba[a] and Shiro Seki[a*]

[a] Graduate School of Applied Chemistry and Chemical Engineering, Kogakuin University, 2665-1 Nakano-machi, Hachioji, Tokyo 192-0015, Japan

[b] Graduate School of Science and Technology, Niigata University, 8050 Ikarashi, 2-no-cho, Nishi-ku, Niigata 950-218, Japan

[c] National Institute of Advanced Industrial Science and Technology (AIST), 1-1-1 Umezono, Tsukuba, Ibaraki 305-8568, Japan

[d] Department of Chemistry and Biotechnology, Yokohama National University, 79-5 Tokiwadai, Hodogaya-ku, Yokohama, Kanagawa 240-8501, Japan

[*] Corresponding author: shiro-seki@cc.kogakuin.ac.jp

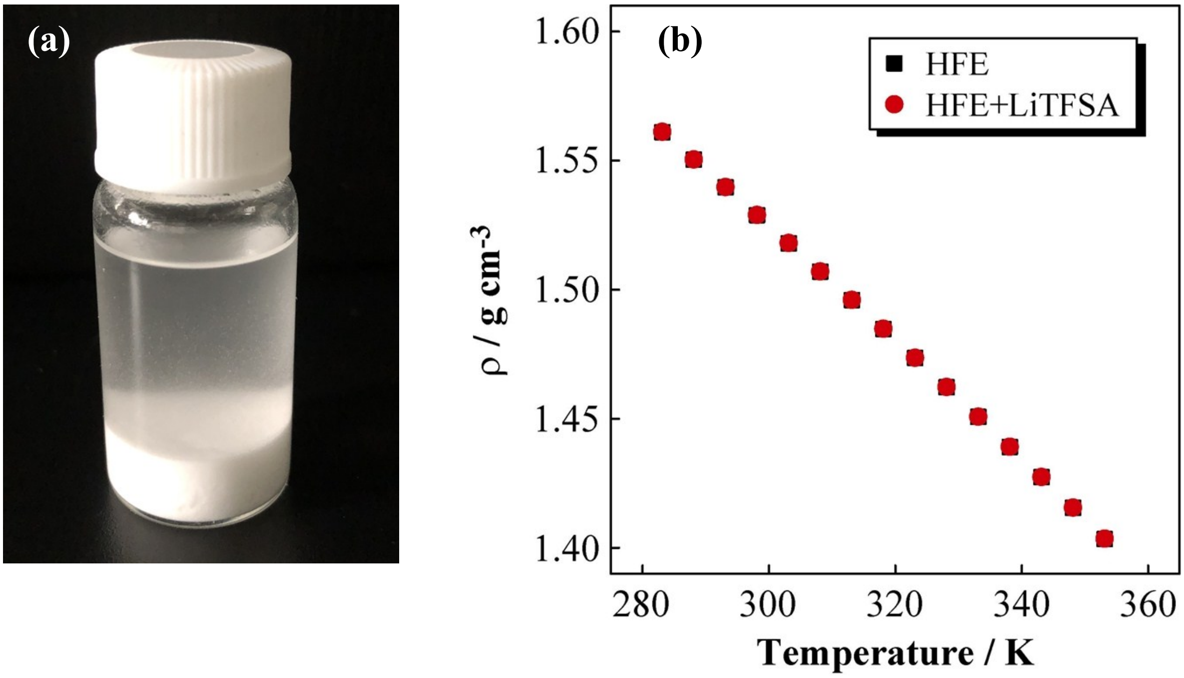

Figure S1 Appearance of LiTFSA and HFE mixture (a) and densities of their supernatant liquid (b).
